# Incoherent Radar Imaging for Breast Cancer Detection and Experimental Validation against 3D Multimodal Breast Phantoms

**DOI:** 10.3390/jimaging7020023

**Published:** 2021-02-01

**Authors:** Antonio Cuccaro, Angela Dell’Aversano, Giuseppe Ruvio, Jacinta Browne, Raffaele Solimene

**Affiliations:** 1 Dipartimento di Ingegneria, Università degli Studi della Campania Luigi Vanvitelli, 81031 Aversa, Italy; antoniocuccarox@gmail.com (A.C.); angela.dellaversano@gmail.com (A.D.); 2 School of Medicine, National University of Ireland Galway, Galway 8, Ireland; giuseppe@endowave.ie; 3 Endowave Ltd., Galway, Ireland; 4 Department of Radiology, Mayo Clinic, Rochester, MN 55905, USA; Browne.Jacinta@mayo.edu; 5 Medical Ultrasound Physics and Technology Group, School of Physics and Clinical & Optometric Sciences, IEO, FOCAS, Technical University Dublin, Dublin 8, Ireland; 6 Consorzio Nazionale Interuniversitario per le Telecomunicazioni-CNIT, 43124 Parma, Italy; 7 Indian Institute of Technology, Madras, Chennai 600036, India

**Keywords:** microwave imaging, incoherent imaging, clutter rejection, breast cancer detection

## Abstract

In this paper we consider radar approaches for breast cancer detection. The aim is to give a brief review of the main features of incoherent methods, based on beam-forming and Multiple SIgnal Classification (MUSIC) algorithms, that we have recently developed, and to compare them with classical coherent beam-forming. Those methods have the remarkable advantage of not requiring antenna characterization/compensation, which can be problematic in view of the close (to the breast) proximity set-up usually employed in breast imaging. Moreover, we proceed to an experimental validation of one of the incoherent methods, i.e., the I-MUSIC, using the multimodal breast phantom we have previously developed. While in a previous paper we focused on the phantom manufacture and characterization, here we are mainly concerned with providing the detail of the reconstruction algorithm, in particular for a new multi-step clutter rejection method that was employed and only barely described. In this regard, this contribution can be considered as a completion of our previous study. The experiments against the phantom show promising results and highlight the crucial role played by the clutter rejection procedure.

## 1. Introduction

Global statistics have demonstrated that breast cancer is the most frequently diagnosed invasive cancer and the leading cause of death due to cancer among female patients [1]. In recent years the incidence of breast cancer in developed countries has continued to rise; but at same time, the rate of mortality has undergone a substantial decline [2]. This is due to the improvements in medical cancer treatment and in the implementation of screening programs as well as to improved imaging techniques [3].

As shown in [4], and as can be naturally expected, the survival of a patient is strongly determined by the stage of the disease at the time the treatment starts. Therefore, early diagnostics is crucial. This requires further improvements of the capabilities of current diagnostic modalities. In addition, over the last few years, this has steered efforts towards the development of new imaging modalities with the aim of supplementing the ones currently employed in the clinical practice.

Current conventional imaging modalities are X-ray mammography, digital breast tomo-synthesis, ultrasound and magnetic resonance imaging (MRI), with mammography actually being the golden standard in breast cancer imaging [5]. Among new imaging modalities, in this paper we focus on microwave breast imaging (MBI). Microwave imaging has triggered a great deal of research over the last decades because it offers a number of potential advantages related to the use of non-ionizing radiation, it does not require to compress the breast and requires a relatively cheap technology [6]. All these features along with the progress achieved in this field [7], show that MBI is actually a “promising imaging modality” [8,9].

Many algorithms for microwave imaging have been tailored for breast diagnostics [10]. Some of them reconstruct a 3D volume; others are based on a sliced approach and, for example, they reconstruct repeated coronal slices of the breast and thus reduce the imaging algorithm complexity and accelerate image reformatting [11]. In any case, microwave breast imaging entails solving a non-linear ill-posed inverse scattering problem since diffraction effects cannot be ignored as in X-ray tomography.

Microwave imaging algorithms can be coarsely grouped in two broad categories, depending on the way non-linearity is dealt with.

When the aim is to reconstruct the dielectric/conductivity profile of the breast tissue under examination, “quantitative” algorithms must be adopted. In these cases, the non-linearity of the problem must be taken into account and the reconstructions are basically achieved by iterative optimization procedures that try to minimize some cost function of the misfit between the available data and the model ones [12]. As such, non-linear inversions are generally computationally very intensive [13] and can suffer from convergence and reliability problems due to false solutions [14]. However, we need to mention that some hybrid approaches, that exploit a priory information provided by other modality, can help mitigate these issues.

The imaging problem is drastically simplified if the imaging method is based on linearized scattering models [15]. In this case, imaging results into linear procedures that are robust and computationally effective. However, only qualitative information can be obtained. Indeed, the corresponding images are more like hot maps where strong inhomogeneities are highlighted. Therefore, linear methods can be conveniently employed if the main objective is to detect and localize targets with a significant dielectric contrast as compared to the surrounding background tissues.

Linear imaging methods are commonly addressed as radar approaches and are the ones we are concerned with in this contribution.

Among the linear methods, beam-forming (BF) is probably the most popular in MBI. Basically, it consists in time-shifting the signals received over the measurement aperture in order to isolate signals scattered from (and hence to focus at) a particular synthetic focal point belonging to the spatial area to be imaged [16]. The BF approach is attractive for the excellent compromise between the achievable performance and the procedure complexity. In [17] the classical delay and sum (DAS) beam-former is used for breast cancer imaging, but many different versions of DAS beam-former have been employed and proposed in literature. For example the delay multiply and sum (DMAS) beam-former is proposed in [18] and the enhanced DAS (EDAS) beam-former in [19]. Besides BF, many other linear inversion methods can be found in literature. For example, a number of linear inversion methods that rely on the spatial spectral representation of the solutions of the wave equation have been developed in different applicative contexts [20]. Among them we mention the range migration [21], the Stolt migration [22], the wave-interference migration [23] and the Holographic Imaging (HI) [24]. These methods are very appealing since their implementation requires computing a Fourier transformation [25,26] which can be effectively achieved via a Fast Fourier Transform (FFT) algorithm. While the latter typically requires the scattered field data to be collected over a planar (rectilinear for 2D cases) measurement aperture, since the Cartesian spatial coordinates naturally match the spatial Fourier transform setting, the extension to deal with circular configurations (more suitable for breast imaging) was previously pursued, for example, in [27].

A detailed analytical comparison between beam-forming and holographic methods has been carried out (for a scattering scenario pertinent to breast imaging) in [28], where the role of critical parameters, such as the operating frequency range, the number of scatterers and data discretization, was considered. Instead, in [29] it is shown that all these methods are variants of the so-called generalized holography.

As remarked above, linear methods restrict the imaging stage to a mere detection and localization of strong in-homogeneities. However, even in light of this reduced task and under the simplified linear framework, the imaging problem is still extremely difficult for a number of reasons. One of the issue, is that all the previous methods “coherently” combine data collected at different frequencies. Therefore, the achievable performance is negatively affected by frequency dispersion of breast tissues (which are unknown or known with a considerable degree of uncertainty and vary from patient to patient) as well as by the antenna frequency response, which is hard to predict because it is in close proximity to breast. As shown in [30,31], this drawback can be mitigated by employing non-coherent imaging strategies. In particular in those papers we introduced and compared incoherent versions of beam-forming and MUSIC [32] (I-MUSIC) and showed that the performance remains stable by using different types of antennas although they were non-characterized, i.e., their frequency responses were not estimated nor enclosed in the model upon which the algorithms were based.

Another crucial aspect is the clutter that generally overwhelms the relatively weak signal coming from the cancer targets. Accordingly, before obtaining the image, data must be first processed in order to reduce the clutter due to the antenna’s internal reflection, the skin interface and other non-tumor breast tissues. To this end, we employed a hybrid clutter removal method [33].

In this contribution we will give a quick review of the incoherent methods and detail the clutter mitigation procedure that was used, but actually not described, in [33]. In particular, the achievable cancer detection is checked by using experimental data collected by employing the multi-modal breast phantom developed in [33]. Accordingly, this paper focuses more on the image process and can be considered as a companion paper of [33] which, instead, mainly considered the development of the breast phantom and its tissue characterization for different imaging modalities.

## 2. Ideal Scattering Configuration and Beam-Forming

**Figure 1:**
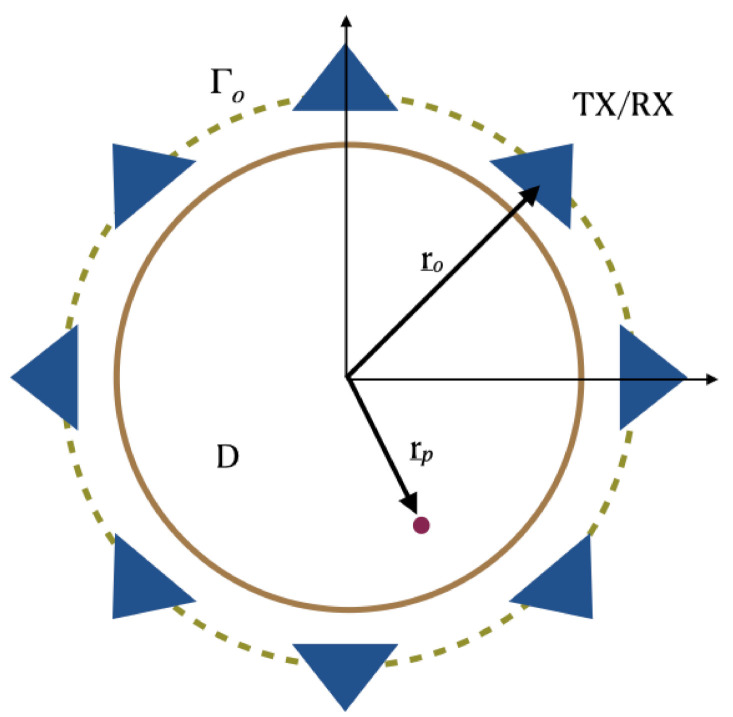
Pictorial view of the scattering scene. Invariance is assumed along the *z*-axis.

In order to introduce the notation and to more easily describe the incoherent imaging methods we consider an idealized scattering scenario. More specifically, the scattering problem is considered for a two-dimensional scalar configuration (see Figure 1). Here, invariance is assumed along the *z*-axis and the electromagnetic incident field has a transverse magnetic TM polarization.

According to the measurement set-up commonly used for breast imaging, sensors are assumed to be located over a circle which is in close proximity and embodies the scattering region *D*. The position of each sensor is identified by the vector ${\underline{r}}_{o}\in \Gamma_{o}$, $\Gamma_{o}$ being the circular measurement curve. The scattered signals are assumed to be collected only at the same position as the transmitter, while the latter can assume different positions over the circle in order to synthesize the measurement aperture. Hence, a multimonostatic configuration is considered. Note that, while the multimonostatic setting is by far the most common, more complex multiview/multistatic sensors’ arrangement are often employed: these configurations are not considered herein.

As far as the background medium is concerned, it is assumed to be homogeneous and lossless. Of course, this does not match with realistic breast structures which consist of many layers of different materials that can have even articulated boundaries. Nonetheless, because the breast structure is actually unknown, and for the sake of simplicity, during the image stage an equivalent homogeneous background medium is usually considered.

According to the previous assumptions and under Born approximation [15], the scattered field in the frequency domain is given as:

(1) $$E_{S}\left(\omega ,{\underline{r}}_{o}\right)={\left(\frac{\omega }{2v}\right)}^{2}P\left(\omega \right)\int_{D}\;G^{2}\left(\omega /v,{\underline{r}}_{0},\underline{r}\right)\chi \left(\underline{r}\right)d\underline{r}$$

where *D* is the spatial region under investigation, ω is the angular frequency and *v* the background propagation speed. Moreover, $E_{S}\left(\omega ,{\underline{r}}_{o}\right)$ is the scattered field data, P(ω) is the temporal Fourier spectrum of the transmitted pulse, $G\left(.\right)=1/4jH_{0}^{\left(2\right)}\left(\cdot \right)$ is the two-dimensional scalar background Green function, with $H_{0}^{\left(2\right)}\left(\cdot \right)$ being the Hankel function of second kind and zero order. Finally, $\chi (\underline{r})$ is the so-called contrast function which describes the scatterers in terms of their shape and electromagnetic parameters. Note that in general $\chi (\underline{r})$ is also frequency dependent but here such a dependence has been neglected. In particular, exploiting the asymptotic expansion of the Hankel function, i.e., $H_{0}^{\left(2\right)}\left(x\right)≃\sqrt{2/\pi x}\exp\left[-j(x-\pi /4)\right]$), Equation (1) can be recast as:

(2) $$E_{S}\left(\omega ,{\underline{r}}_{o}\right)=\left(j\omega /2\pi v\right)P\left(\omega \right)\int_{D}\frac{\exp\left(\frac{-j2\omega }{v}\left|{\underline{r}}_{o}-\underline{r}\right|\right)}{|{\underline{r}}_{o}-\underline{r}|}\chi \left(\underline{r}\right)d\underline{r}$$

In practice, the quantity that is actually measured is not the scattered field but rather the system scattering parameters. This basically entails taking in account the antenna frequency response. Accordingly, (2) modifies as:

(3) $$S\left(\omega ,{\underline{r}}_{o}\right)=\left(j\omega /2\pi v\right)\tilde{P}\left(\omega \right)\int_{D}\frac{\exp\left(\frac{-j2\omega }{v}\left|{\underline{r}}_{o}-\underline{r}\right|\right)}{|{\underline{r}}_{o}-\underline{r}|}\chi \left(\underline{r}\right)d\underline{r}$$

where $\tilde{P}\left(\omega \right)=H^{2}\left(\omega \right)P\left(\omega \right)$ now takes into account the squared (because the antenna acts as TX and RX) antenna response assumed to be solely dependent on the frequency ω and $S(\omega ,{\underline{r}}_{o})$ are the scattering measurements.

In order to introduce the beam-forming method, it is convenient to consider the time domain version of Equation (3). Hence, by Fourier transforming with respect to ω, we obtain the time domain scattering measurements as:

(4) $$s\left(t,{\underline{r}}_{o}\right)=\int_{D}\tilde{p}\left[t-\tau \left({\underline{r}}_{o},\underline{r}\right)\right]\chi \left(\underline{r}\right)d\underline{r}$$

where $\tilde{p}\left(.\right)$ is related to the transmitted pulse and is the Fourier transform of $\left(j\omega /2\right)\tilde{P}\left(\omega \right)/\left|{\underline{r}}_{0}-\underline{r}\right|$ and $\tau \left({\underline{r}}_{o},\underline{r}\right)=2/v\left|{\underline{r}}_{0}-\underline{r}\right|$ is the round-trip delay.

Generally, the image obtained by the DAS beam-forming is given by:

(5) $$I_{BF}\left(\underline{r}\right)=\int W\left(t\right){\left[\int_{\Gamma_{o}}s\left[t-T\left({\underline{r}}_{o},\underline{r}\right),{\underline{r}}_{o}\right]d{\underline{r}}_{o}\right]}^{2}dt$$

where W(.) is a suitable time window and $T\left({\underline{r}}_{o},\underline{r}\right)=T_{W}-\tau \left({\underline{r}}_{o},\underline{r}\right)$, with $T_{W}=max_{{\underline{r}}_{o},\underline{r}}\left\{\tau \left({\underline{r}}_{o},\underline{r}\right)\right\}$. Accordingly, the received signals are “aligned” at the time instant $T_{W}$ and then summed. In particular, by setting $W\left(t\right)=\delta \left(t-T_{W}\right)$ the reconstruction $I_{BF}$ becomes:

(6) $$I_{BF}\left(\underline{r}\right)={\left[\int_{\Gamma_{o}}s\left[T_{W}-T\left({\underline{r}}_{o},\underline{r}\right),{\underline{r}}_{o}\right]d{\underline{r}}_{o}\right]}^{2}$$

and returning back to the frequency domain (details can be found in [28]):

(7) $$I_{BF}\left(\underline{r}\right)={\left|\int_{\Omega }\int_{{\underline{\Gamma }}_{o}}S\left(\omega ,{\underline{r}}_{o}\right)\exp\left[j\omega \tau \left({\underline{r}}_{o},\underline{r}\right)\right]d{\underline{r}}_{o}d\omega \right|}^{2}$$

Equation (7) is functional to appreciate the difference with the incoherent approach to be shown in the sequel. In addition, it allowed a closed-form derivation of the point-spread function, that is the reconstruction of a point-like target $\chi \left({\underline{r}}_{p}\right)=\delta \left(\underline{r}-{\underline{r}}_{p}\right)$, that permits to evaluate the achievable resolution in terms of the configuration parameters, including the frequency range and the data discretization [28]. In particular, it was shown that the common belief that in order to achieve a finer resolution a wider frequency band is required does not necessarily hold true. Indeed, while this statement is correct for aspect-limited configurations, for the case at hand, where measurements can be taken all around the scattering region (i.e., non-aspect limited configuration, see Figure 1), finer resolution can be obtained by moving a fixed frequency band towards high frequencies. This is an important result which has practical implications since it promotes the use of cheaper hardware and simplifies the antenna design, which does not necessarily have to work on an ultra-wide band. All details can be found in [28].

## 3. Incoherent Image Procedures

As highlighted in (5), the measured scattering parameters depend on the antenna response. Indeed, this enters in shaping the frequency behaviour of $\tilde{P}\left(\omega \right)$ and in general introduces a frequency dependent propagation delay. The latter must be considered while setting the time window W(t) and the alignment time $T_{w}$. This requires near-field antenna characterization/equalization that can be pursued by a suitable set of measurements or numerical simulations. However, as the breast properties change from patient to patient, residual errors still remain. With uncertainty levels as high as the magnitude of the tumor scattered field, the imaging procedure’s robustness is dramatically endangered. This is particularly true for dense breasts as they present lower tumor/healthy-tissue contrast. It can be noted that this drawback arises because frequency data are coherently summed. Therefore, a viable way to mitigate this problem is to devise imaging schemes which do rely on such a coherence and process each frequency data separately. This is the topic addressed in this section.

### 3.1. Incoherent Beam-Forming

Basically, incoherent beam-forming (IBF) is achieved as follows:

(8) $$I_{IBF}\left(\underline{r}\right)=\int_{\Omega }{\left|\int_{\Gamma_{o}}S\left(\omega ,{\underline{r}}_{o}\right)\exp\left[j\omega \tau \left({\underline{r}}_{o},\underline{r}\right)\right]d{\underline{r}}_{o}\right|}^{2}d\omega$$

where the basic difference, with respect to (5), is clearly that data are summed in amplitude along the frequency domain. Of course, it is interesting to elucidate how (8) relates physically (meaning) and in terms of the achievable performance to (5). As shown in [28], the time domain counterpart of (8) is

(9) $$I_{IBF}\left(\underline{r}\right)=\int {\left[\int_{\Gamma_{o}}s\left[t-T\left({\underline{r}}_{o},\underline{r}\right),{\underline{r}}_{o}\right]d{\underline{r}}_{o}\right]}^{2}dt$$

where basically the window function has been removed. Form the achievable performance point of view, in [28] the point-spread function was also analytically derived for the incoherent case and it was found that the main difference with respect to the coherent case is that side-lobes are slightly higher. However, the point-spread function main beams (and hence the resolution) are practically the same. Therefore, the cost to pay while using (8) in place of (5) is that side-lobe reconstruction increases a little bit (of course, the actual increase depends on the configuration parameters, especially by the frequency band) but this is largely rewarded since the need to estimate/compensate the antenna response is avoided.

### 3.2. Discrete Data Setting

In the previous section we implicitly considered the situation where data are collected continuously all around the scattering scene. In practice, the number of data samples must be finite. Accordingly, in this section we recast the previous argument within a discrete data setting which, in turn, is also necessary to introduce the I-MUSIC, as shown below.

Therefore, say ${\underline{r}}_{o1},{\underline{r}}_{o2},\cdots ,{\underline{r}}_{oN_{o}}$$N_{o}$ measurement points taken uniformly over the measurement circle $\Gamma_{o}$ and $\omega_{1},\omega_{2},\cdots ,\omega_{Nf}$ the employed frequencies. In addition, denote as ${\underline{r}}_{1},{\underline{r}}_{2},\cdots ,{\underline{r}}_{N_{s}}$ the coordinates of the pixels that divide the spatial region under test *D*. The finite dimensional (discrete) counterpart of (1) can then be written as:

(10) $$S^{i}\left(\omega_{i}\right)={\left(\omega_{i}/2v\right)}^{2}\tilde{P}\left(\omega_{i}\right)A\left(\omega_{i}\right)b\left(\omega_{i}\right)$$

where

(11) $$S^{i}\left(\omega_{i}\right)={\left[S\left({\underline{r}}_{o1},\omega_{i}\right),S\left({\underline{r}}_{o2},\omega_{i}\right),\cdots ,S\left({\underline{r}}_{oN_{o}},\omega_{i}\right)\right]}^{T}\in C^{N_{o}}$$

is the column vector of the scattering data collected at frequency $\omega_{i}$,

(12) $$b^{i}\left(\omega_{i}\right)={\left[b_{1}\left(\omega_{i}\right),b_{2}\left(\omega_{i}\right),\cdots ,b_{N_{s}}\left(\omega_{i}\right)\right]}^{T}\in C^{N_{s}}$$

is the vector of the pixel scattering coefficients), ${\left(\cdot \right)}^{T}$ denoting the transpose, and $A\left(\omega_{i}\right)\in C^{N_{o}\times N_{s}}$ is the $N_{o}\times N_{s}$ matrix propagator (indeed a discrete version of Equation (1)) whose *n*-th column has the form:

(13) $$A^{n}\left({\underline{r}}_{n},\omega_{i}\right)={\left[G^{2}\left(\omega_{i},{\underline{r}}_{o1},{\underline{r}}_{n}\right),G^{2}\left(\omega_{i},{\underline{r}}_{o2},{\underline{r}}_{n}\right),\cdots ,G^{2}\left(\omega_{i},{\underline{r}}_{oN_{o}},{\underline{r}}_{n}\right)\right]}^{T}$$

where the Green function is the same as in Equation (2). Accordingly, the overall data scattering matrix is:

(14) $$S=\left[S^{1}\left(\omega_{1}\right)\;S^{2}\left(\omega_{2}\right)\;\cdots \;S^{N_{f}}\left(\omega_{N_{f}}\right)\right]\in C^{N_{o}\times N_{f}}$$

Due to this discrete setting, Equation (8) can be particularized as:

(15) $$I_{IBF}\left(\underline{r}\right)=\sum_{m=1}^{N_{f}}{\left|\sum_{l=1}^{N_{o}}S\left(\omega_{m},{\underline{r}}_{ol}\right)\exp\left[j\omega_{m}\tau \left({\underline{r}}_{on},\underline{r}\right)\right]\right|}^{2}$$

where $\underline{r}\in {\underline{r}}_{1},{\underline{r}}_{2},\cdots ,{\underline{r}}_{N_{s}}$. A crucial question to be addressed within the discrete setting is the choice of the minimum number of sensor positions that should be deployed around the scattering scene in order to obtain the same results as the ideal case (i.e., data collected continuously) or at least to avoid aliasing effects that can result in reconstruction crowed by spurious artifacts that can be mistaken for actual targets. In particular, it is shown that to avoid aliasing a sufficient condition is that the number of measurement points be:

(16) $$N_{o}\geq 4k_{max}R_{c}$$

where $k_{max}$ is the wave number corresponding to the highest adopted frequencies and $R_{c}<R$ the radius of the circular investigation domain. Basically, Equation (16) guarantees that data are properly “spatially” sampled for each adopted frequency. However, because of the multifrequency data, and the related mutifrequency reconstruction process, some degree of under-sampling can be tolerable for part of the frequency band. This is because aliasing spurious artifacts are frequency dependent. Thus, their positions change with the frequency. By contrast, the main contribution of the reconstruction always peaks at the actual scatterer’s location. Therefore, even if condition (16) is not satisfied, while summing up different frequency contributions in (15), artifacts tend to be averaged out whereas the main beam (due to scatterer) is not.

### 3.3. Incoherent MUSIC

The starting point is the construction of the correlation matrix for each frequency, that is:

(17) $$R\left(\omega_{i}\right)=S^{i}\left(\omega_{i}\right)S^{iH}\left(\omega_{i}\right)=A\left(\omega_{i}\right)B\left(\omega_{i}\right)A^{H}\left(\omega_{i}\right)$$

where $b^{H}\left(\omega_{i}\right)$ and $A^{H}\left(\omega_{i}\right)$ are the Hermitian vector and matrix of $b(\omega_{i})$ and $A(\omega_{i})$, respectively, and $B\left(\omega_{i}\right)=b\left(\omega_{i}\right)b^{H}\left(\omega_{i}\right)$. According to [32], scatterers can be localized by finding the steering vectors which are orthogonal to the so called noise subspace. This requires computing the eigenspectrum of $R(\omega_{i})$ and the steering vectors which basically consists of the normalized columns of the propagator $A(\omega_{i})$, that is $\mathrm{Sv}\left({\underline{r}}_{n},\omega_{i}\right)=A^{n}\left({\underline{r}}_{n},\omega_{i}\right)/∥A^{n}\left({\underline{r}}_{n},\omega_{i}\right)∥$ being evaluated in correspondence to the trial position ${\underline{r}}_{n}$ within the spatial domain *D*. Hence, scatterers’ positions are identified where the pseudospectrum

(18) $$\phi \left({\underline{r}}_{n},\omega_{i}\right)=\frac{1}{∥P_{N}\left[{\mathrm{Sv}}^{n}\left(\omega_{i}\right)\right]{∥}^{2}}$$

peaks, with $P_{N}\left[\cdot \right]$ being the projection operator onto the noise subspace. However, as shown in [31,34], the correlation matrix is rank deficiency with rank one. Therefore, the scheme to identify the scatterers’ location can be modified defining $P_{N}=\left(I-P_{S}\right)$, with $P_{S}\left[\cdot \right]$ being the projector onto the signal space. Hence, the detection is achievable by adopting the only significant singular vector associated to the signal subspace.

Note that Equation (18) refers to single frequency data. Multiple frequencies can be incoherently combined [35] giving rise to

(19) $$\Phi_{I-MUSIC}\left({\underline{r}}_{n}\right)=\prod_{i=1}^{N_{f}}\phi \left({\underline{r}}_{n},\omega_{i}\right)$$

Eventually, (19) is the proposed as algorithm for cancer detection.

### 3.4. Numerical Comparison

**Figure 2:**
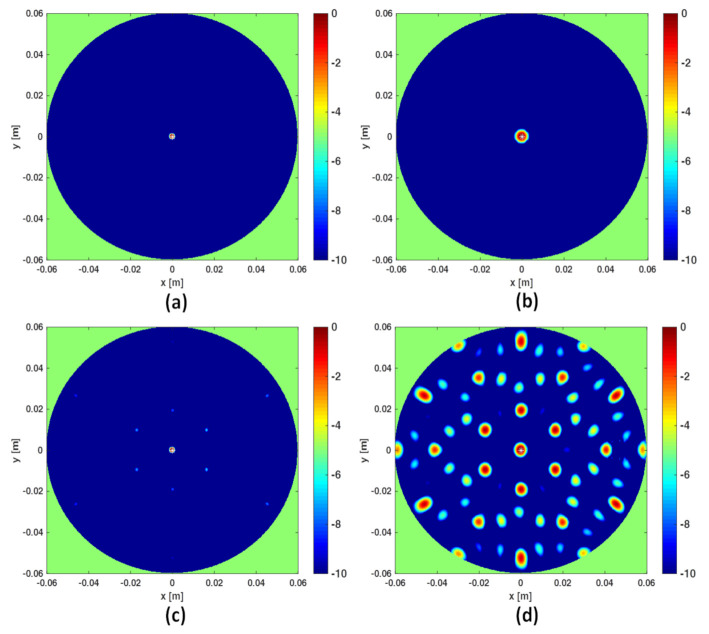
Comparing I-MUSIC and beam-forming (BF) for single frequency data. The left column refers to I-MUSIC; the right one to BF. In panels (**a**,**b**) $N_{o}=49$ whereas in panels (**c**,**d**) $N_{o}=7$.

In this section a numerical comparison between the I-MUSIC and the beam-forming strategies is shown. Initially, single frequency (f=3 GHz) data are considered and a background medium with $\epsilon_{r}=9$. The investigation domain *D* is assumed to be a circle of radius $R_{c}=6$ cm whereas measurements are taken over a concentric circle of radius *R* slightly greater than $R_{c}$. A point-like target is located in the centre of *D*. For the case at hand, Equation (16) suggests $N_{o}>45$ to avoid artefacts. The reconstructions corresponding to this case are shown in panels (a) and (b) of Figure 2. Note that at single frequency there is no difference between BF and IBF. Accordingly, in Figure 2 we just refer to beam-forming. When the number of points is lowered, spurious artefacts actually corrupt the reconstructions. In particular, the bottom panels of the same figure depict the reconstructions when the number of sensors is reduced by seven times. These results are perfectly consistent with the theory developed in [28,34].

**Figure 3:**
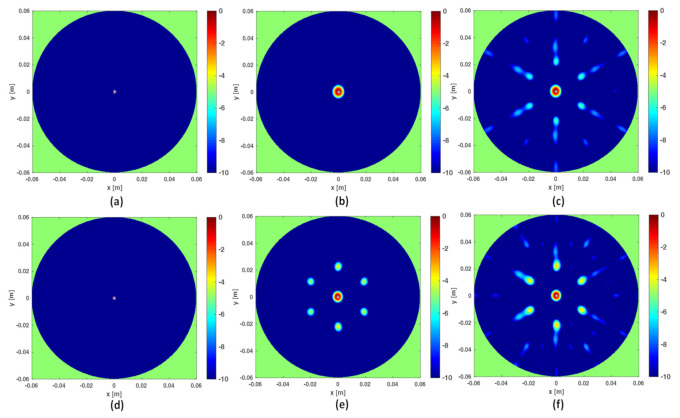
Illustrating the role of the frequency band. In all the reconstructions only $N_{o}=7$ sensors are considered. In the top panels the frequency band is [1,3] GHz, in the bottom panels the frequency band is reduced to [2,3] GHz. Finally, (**a**,**d**) refer to I-MUSIC, (**b**,**e**) to BF and (**c**,**f**) to IBF.

As mentioned above, frequencies are a good ally to mitigate artifacts when the data are under-sampled. This can be observed in Figure 3 where the three reconstruction schemes, i.e., I-MUSIC, BF and IBF, are compared for the same cases as in panels (c) and (d) of Figure 2 but by considering two different frequency bands. As can be seen, the frequency band greatly helps in reducing artefacts. In addition, as expected and according to previous discussion, IBF presents higher side-lobe levels than BF. Furthermore, I-MUSIC outperforms BF schemes since it allows for a more sharper target localization and better resilience to aliasing. Therefore, I-MUSIC is the method that has been selected for undergoing the experimental validation reported in the sequel.

## 4. Experimental Analysis

As mentioned in the introduction, this paper can be regarded as a companion paper of [33]. In that paper, we mainly focused on the design, construction and characterization of the breast phantom; microwave imaging algorithms were not described at all. While the detection algorithm was actually the I-MUSIC that we have already described in previous contributions (and whose main ingredients have been briefly recalled above in conjunction to the comparison with more classical BF methods) the clutter rejection algorithm deserves a more in-depth description. Therefore, the program for this section is to first briefly report about the measurement set-up and the breast phantom and then to move on to a detailed description of the clutter rejection method. Finally, a few experimental reconstructions are used to show the effectiveness of the I-MUSIC + de-cluttering procedure.

### 4.1. Measurement Set-Up

**Figure 4:**
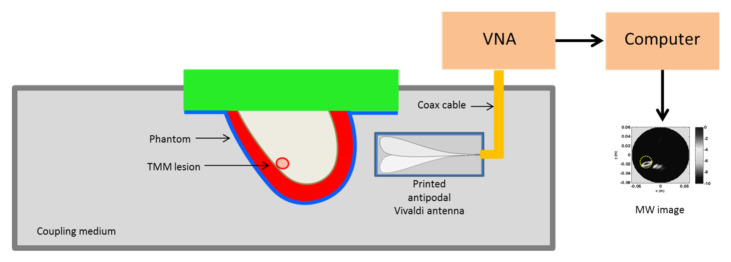
Schematic diagram showing the MBI scanning setup. The system antenna + phantom is immersed in a coupling medium. The antenna is connected to a Vector Network Analyzer (VNA) scanning the phantom at a fixed height in multimonostatic configuration. This allows collecting data for a single coronal slice.

The pictorial view of the measurement set-up is shown in Figure 4 and basically coincides with the measurement scheme adopted in [36]. A breast phantom was scanned by an antipodal Vivaldi antenna in the frequency range [0.5–5] GHz connected to a VNA. In particular, at a given height the antenna rotated around the phantom (with a 5${}^{\circ }$ angular step) in order to synthesize a multimonostatic configuration (i.e., $T_{X}$ and $R_{X}$ were co-located) for a total 72 scanning positions. In general, data collected at different heights can be simultaneously employed to get a 3D reconstruction. However, here we exploited the sliced approach. The phantom and the antenna were immersed within a coupling medium with relative dielectric permittivity equal to 12. This was done for antenna miniaturization purposes and to reduce the dielectric discontinuity from the antenna side to the breast, which can hinder microwave energy penetration [35]. Accordingly, such a value of the dielectric permittivity was used to define the equivalent homogeneous reference background medium which was used to build up the scattering model upon which the detection algorithm was based. No information concerning the phantom nor the antenna response (which was not estimated or compensated) was exploited in the following image stage.

### 4.2. Breast Phantom

In [33] we developed multimodal anthropomorphic breast phantoms suitable for evaluating the imaging performance of microwave imaging in comparison to the established diagnostic imaging modalities of Magnetic Resonance Imaging, Ultrasound, Mammography and Computed Tomography. In that study, the aim was to build a bridge between the numerical simulation environment and a more realistic diagnostic scenario. To this end, the constructed anthropomorphic phantoms mimic breast tissues in terms of their heterogeneity, anatomy, morphology, and mechanical and dielectric characteristics and reproduce different healthy and pathologic tissue types for each of the modalities, taking into consideration the differing imaging and contrast mechanisms for each modality. In that study, two phantoms were developed: the phantom (named as ‘Phantom A’) had a simple and less morphologically accurate interface between mammary fat and fibroglandular tissue; the second (’Phantom B’) had a more relevant complex fat and fibroglandular interface. Both were extracted from real patient MRI datasets. Apart from the different morphological structure, the phantoms had the same five different tissue-mimicking materials: skin, subcutaneous fat, fibroglandular tissue, pectoral muscle and tumor. The phantoms’ construction used non-toxic materials, and they were inexpensive and relatively easy to manufacture. Both phantoms were characterized and scanned using conventional modalities (MRI, US, mammography and CT). The details concerning all the steps required for their manufacturing, characterization and imaging can be found in [33]. Their MRI coronal slices are reported below and highlight the different tissue mimicking layers as well as the tumor. In particular, it can be seen that in both cases the tumor was located inside the fibroglandural structure. The tissue dielectric permittivity and the conductivity of the different tissues are reported in Figure 1 of [33] which shows that the dielectric contrast between tumor and fibroglandural tissue was at best 1.2:1 (hence extremely low) within the frequency band [0.5,4] GHz.

### 4.3. Clutter Rejection Algorithm

In order to obtain the reconstruction, in our case a single coronal slice, before the imaging stage, data had to be properly processed in order to reduce clutter disturbances that arose from the antenna internal reflections, the skin interface, and other breast tissues. As the clutter tends to mask the informative signal, it needed to be reduced before the image construction procedure was run. Different clutter rejection methods have been proposed in the literature. For example, in [36] a hybrid artefact removal algorithm for microwave imaging is used, while in [37] some of the most common algorithms used in Through Wall Imaging (TWI) applications were compared, including the simplest average trace subtraction strategy. In this paper, a new method for “extracting” the useful signal is proposed. It was based on a two-step entropy computation and a subspace projection stage. The first entropy step was used to set a time-gating procedure in order to remove the strong antenna’s internal reflections and skin contribution; the second one was instead used to select the subset of sensors’ positions where tumor contribution is stronger. Finally, a subspace projection procedure [38] was aimed at mitigating contributions due to breast inhomogeneities. After mitigating the clutter, I-MUSIC was employed to obtain the image.

For convenience we rewrote the scattering data matrix as follows:

(20) $$S=\left[\begin{array}{c} S_{1}\\ S_{2}\\ \vdots \\ S_{N_{o}} \end{array}\right]$$

where $S_{i}$ are the rows of S and hence vectors whose indexes range over the frequencies, i.e., $S_{i}\in C^{N_{f}}$. In order to compute the time gate, the first step is to transform the rows of S in time domain. Accordingly, upon applying an IDFT routine, we get:

(21) $$s=\left[\begin{array}{c} s_{1}\\ s_{2}\\ \vdots \\ s_{N_{o}} \end{array}\right]$$

with $s\in C^{N_{o}\times N_{t}}$ being the time domain version of S and $N_{t}$ is the number of retained time domain samples. Hence, the rows of s are basically the time-traces (A-scan in usual radar literature) collected over the different antenna positions. Note that if data had already been collected in time domain, this step would have not be necessary.

In Reference [39], an entropy-based metric was used to discriminate between clutter and target signals. The same idea was adopted here to seek a suitable time-gating. Accordingly, normalized time traces, ${\tilde{s}}_{n}$, were constructed whose entries are given by:

(22) $${\tilde{s}}_{n}\left(t_{m}\right)=\frac{|s_{n}\left(t_{m}\right)|}{\sum_{l=1}^{N_{o}}\left|s_{l}\left(t_{m}\right)\right|}\;\forall t_{m}=t_{1},\cdots ,t_{N_{t}}$$

where $s_{n}\left(t_{m}\right)$ is just the m-th entry of $s_{n}$, i.e., the n-th time trace. Now, ${\tilde{s}}_{n}\left(t_{m}\right)\geq 0$ and $\sum_{n=1}^{N_{o}}{\tilde{s}}_{n}\left(t_{m}\right)=1$, $\forall t_{m}$. Therefore, for each instant of time, the vector of the normalized data could be assimilated to a probability density function. This observation suggested introducing the entropy measure as

(23) $$\epsilon_{s}\left(t_{m}\right)=-\sum_{n=1}^{N_{o}}{\tilde{s}}_{n}\left(t_{m}\right)\log\left[{\tilde{s}}_{n}\left(t_{m}\right)\right]$$

**Figure 5:**
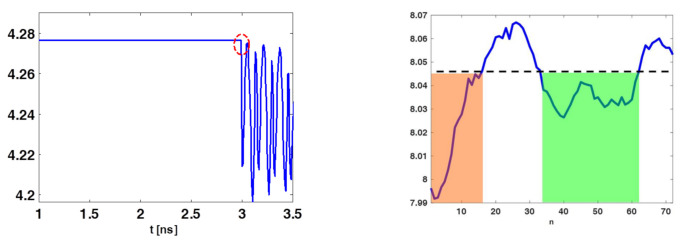
Illustrating entropy behaviour for the case of phantom B. The (**left**) panel shows the entropy $\epsilon_{s}\left(t_{m}\right)$ and the red dashed circle identifies the time-gating value (3 ns). The (**right**) panel shows ${\hat{\epsilon }}_{s}$, the orange and green shaded regions highlights the set of sensors’ positions whose data can be retained.

It was expected that $\epsilon_{s}$ was high for those instants of time for which the received signals were similar across the different spatial acquisitions. Of course, this occurred when the antenna was receiving its internal reflections or the skin contribution. Figure 5 (left panel) shows a typical entropy behavior obtained for the collected data. As can be observed, $\epsilon_{s}$ was nearly constant and high until the time $t_{m_{min}}$ (marked by the dashed red circle), where the entropy attained its first abrupt change compared to its maximum value $\log(N_{o})$. According to previous discussion, signals coming from the phantom should start to be received only beyond $t_{m_{min}}$. Signals before such an instant must be discarded. This can be enforced by adopting a time-gating with a time-windowing that removes signals for $t_{m}<t_{m_{min}}$, that is:

(24) $$s_{Wn}\left(t_{m}\right)=W\left(t_{m}\right)s_{n}\left(t_{m}\right)$$

with

(25) $$W\left(t_{m}\right)=\left\{\begin{array}{cc} 0 & \mathrm{if}\;t_{m}\leq \;t_{m_{min}}\\ 1 & \mathrm{elsewhere} \end{array}\right.$$

After time gating, the scattering data matrix was denoted as:

(26) $$s_{W}=\left[\begin{array}{c} s_{W1}\\ s_{W2}\\ \vdots \\ s_{WN_{o}} \end{array}\right]$$

and a further entropy-based windowing was applied. More in detail, $s_{Wn}$ were further normalized as follows:

(27) $${\hat{s}}_{Wn}\left(t_{i}\right)=\frac{|s_{Wn}\left(t_{i}\right)|}{\sum_{l=1}^{N_{t}}\left|s_{Wn}\left(t_{l}\right)\right|}$$

Note that now each time trace underwent a different normalization with the normalizing factor being provided by the summation of the magnitude of its time samples. Once again, it follows that ${\hat{s}}_{Wn}\left(t_{i}\right)>0$ and $\sum_{i=1}^{N_{t}}{\hat{s}}_{Wn}\left(t_{i}\right)=1$, for each sensor’s position. Hence, the $N_{t}$-dimensional vectors of the normalized windowed A-scans could be assimilated as above to a probability density function and the corresponding entropy can be computed as follows:

(28) $${\hat{\epsilon }}_{s}\left({\underline{r}}_{on}\right)=-\sum_{l=1}^{N_{t}}{\hat{s}}_{Wn}\left(t_{l}\right)\log\left[{\hat{s}}_{Wn}\left(t_{l}\right)\right]$$

where ${\underline{r}}_{on}$ is the *n*-th sensor’s position index. The rationale behind this further entropy step is the following. If data at a given position are mainly contributed by clutter and noise then a relatively high level of entropy is expected. This is because the signal magnitude along time should be nearly the same. Instead, when the target significantly contributes then the entropy should decrease. Accordingly, the subset of measurements that effectively “see” the target can be roughly identified by looking for where ${\hat{\epsilon }}_{s}\left({\underline{r}}_{on}\right)$ is below a threshold value. Say $n_{i}$ and $n_{s}$ indicate the positions in between ${\hat{\epsilon }}_{s}\left({\underline{r}}_{on}\right)$ is below the given threshold, then a windowing is applied to keep only the time traces collected over the positions indexed between $n_{i}$ and $n_{s}$. The entropy behaviour reported in Figure 5 (right panel) illustrates the previous discussion. In particular, only data whose positions belonged to the orange and green shaded regions should be retained during the image formation (the threshold was chosen heuristically). In particular, in the following reconstructions only sensors relative to the green zone were retained. Eventually, the second entropy step resulted in a selection of some of the rows of $s_{W}$ reported in (26), so that the data to be used were:

(29) $${\hat{s}}_{W}=\left[\begin{array}{c} s_{Wn_{i}}\\ s_{Wn_{i+1}}\\ \vdots \\ s_{Wn_{n_{s}}} \end{array}\right]$$

These two-step entropy strategies allowed us to select the time-gating to apply to each time trace and to select the sub-set of data (across the different sensor positions) to employ in the reconstruction stage. However, this did not yet ensure that in the remained traces there was only the tumor signal. On the contrary, the latter could still be overshadowed by clutter due to the internal inhomogeneity of the breast. To mitigate this clutter residue, it was convenient to return back into the frequency domain (even because the detection algorithm worked in such a domain) by a DFT routine. Hence, (30) becomes:

(30) $${\hat{S}}_{W}=\left[\begin{array}{c} S_{Wn_{i}}\\ S_{Wn_{i+1}}\\ \vdots \\ S_{Wn_{n_{s}}} \end{array}\right]$$

which is a ${\bar{N}}_{o}\times N_{f}$ matrix, with ${\bar{N}}_{o}$ being the actual number of measurement positions. For the case at hand ${\bar{N}}_{o}=n_{s}-n_{i}$. Then, it was reasonable to assume that clutter magnitude was higher than tumor signals. Accordingly, a clutter-rejection subspace-based technique was adopted. In particular, the retained scattering matrix was first expressed in terms of its singular value decomposition (SVD):

(31) $${\hat{S}}_{W}=U\Lambda V^{H}$$

where U and V are unitary matrices containing the left and the right singular functions, respectively, and Λ is a diagonal matrix containing the singular values $\lambda_{1},\lambda_{2},\cdots ,\lambda_{P}$, in decreasing order, with *P* = $\min[{\bar{N}}_{o},N_{f}]$. Clutter could then be mitigated by disregarding the projection of the scattering matrix ${\hat{S}}_{W}$ onto the singular functions corresponding to the highest singular values. The number of projections to discard generally required a priori information on the clutter, which were in general not available. However, as shown in [35], a conservative choice is to discard the projections of the scattering matrix over the singular functions corresponding to the first or the first two highest singular values. Accordingly, the final de-cluttered data matrix was obtained as:

(32) $$S_{d}=\sum_{l=2\;or\;l=3}^{P}\lambda^{l}u^{l}{\left(v^{l}\right)}^{H}$$

with $u_{l}$ and $v_{l}$ being the *l*-th column vectors of U and V, respectively. Eventually, $S_{d}$ is the data matrix passed to the I-MUSIC stage.

### 4.4. Reconstruction Results

According to the sliced approach mentioned above, data collected at different heights were singularly processed to get the corresponding coronal slice reconstructions. In the sequel we just show only those ones obtained from data collected at the height corresponding to the centre of the tumor. In particular, although data were collected within the frequency band [0.5,5] GHz, in the following reconstructions only the band $B_{w}=\left[1,3\right]$ GHz was exploited.

The rationale under the following examples is to appreciate the role played by the various steps the clutter rejection method consists of as well as the number of frequencies to be employed in the reconstructions.

**Figure 6:**
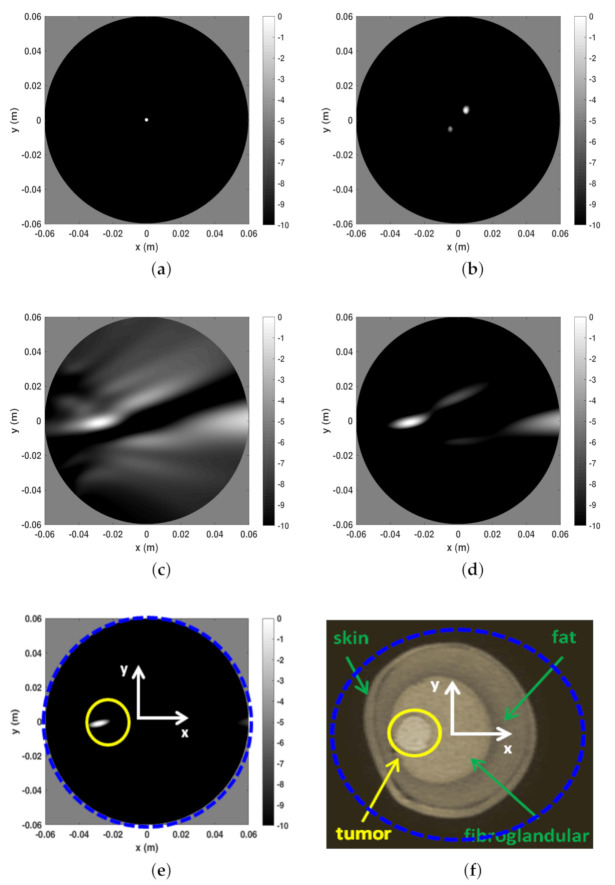
Reconstructions and MR image for phantom A. (**a**) Reconstruction with only time-gating, $N_{f}=20$. (**b**) Reconstruction with time-gating + rejection of the first SVD projection of the scattering matrix, $N_{f}=20$ (**c**) Reconstruction with time-gating + sensor selection + rejection of the first SVD projection of the scattering matrix, $N_{f}=20$. (**d**) Reconstruction with time-gating + sensor selection + rejection of the first SVD projection of the scattering matrix, $N_{f}=40$. (**e**) Reconstruction with time-gating + sensor selection + rejection of the first SVD projection of the scattering matrix, $N_{f}=100$. (**f**) MR coronal slice image of Phantom A. In particular, in panel (**f**) the blue dashed circle indicates the circular boundary of the spatial region within which the reconstructions reported in the other panels have been achieved. This is highlighted even in panel (**e**). Moreover, in the latter, the yellow circle denotes the tumor location and size.

The first example is shown in Figure 6 and refers to Phanton A whose MRI is reported in panel (f) to appreciate the breast internal morphology and for comparison purposes with respect to the microwave imaging. In that figure a blue dashed circle is also reported which identifies the spatial region used to perform the reconstructions. As can be seen, such a spatial region was larger than the the phantom coronal slice. In panels (a) to (e) the I-MUSIC indicator is reported. In particular, in panel (a) to (c) only 20 frequencies, uniformly taken within $B_{w}$, were exploited. More in detail, panel (a) shows the image obtained by pre-processing the data through only the time-gating procedure, by setting the time-gating at $t_{m_{min}}$ = 3 ns according to the first entropy step described above. As can be seen, the reconstruction just returned a hot spot roughly located at the centre of the imaging area. Since I-MUSIC tends to peak at the centre of targets, this means that only time-gating data were not enough to detect the tumor as data still appeared as if produced by a target whose equivalent centre was roughly in the centre of the scene. In panel (b), in order to improve clutter rejection, the subspace approach (achieved by discarding just the first projection of the scattering data matrix) was added to the time-gating. Once again, reconstruction peaks did not match with the expected tumor location, meaning that data were still dominated by a strong clutter contribution. Finally, in panel (c) we also enclosed the sensor selection procedure according to the second entropy computation described in the previous section. In particular, the 2D image was obtained by exploiting only the sensors whose index ranges from $n_{i}=28$ to $n_{s}=56$, which correspond to an angular coverage between $135^{\circ }$ and $275^{\circ }$. For the case at hand, hence, the entropy procedure selected the part of the measurement circular line that is closer to the tumor. This is consistent with the adopted multimonostatic configuration since the scattered field data were collected only in reflection mode. As can be seen, now the tumor was clearly detected and this highlighted that the sensor selection (often overlooked in literature) was a crucial step in addressing imaging in a highly cluttered scenarios. Panel (c) also shows that the image was strongly populated by secondary lobe contributions. This might be due to the employed reduced number of sensors (arising from the second entropy step) which mainly impacted the side lobe structure of the point-spread function. According to the discussion reported above concerning the role of the number of frequencies in mitigating aliasing artefacts, and in general side lobe structure, the quality of reconstruction can be improved by using more frequencies. This was actually the case as can be appreciated looking at panels (d) and (e), where the number of frequencies was increased to $N_{f}=40$ and $N_{f}=100$, respectively. In particular, panel (e) shows an extremely clear tumor detection. Additionally, in that panel we marked through a yellow circle the actual tumor position.

A few comments are in order concerning the obtained reconstruction matching to what I-MUSIC is expected to return. As explained above, I-MUSIC is a radar approach and hence, as such, it is mainly asked to provide tumor detection and rough location. Therefore, it does not aim at reproducing the breast tissue profile as MRI does. At the microwave regime, this task can be attempted by exploiting more sophisticated reconstruction methods that perform the non-linear inversion. Ideally, $\Phi_{I-MUSIC}$ allows for very sharp tumor location (as compared to BF), as shown in the illustrative numerical examples reported above. However, because of uncertainties, clutter residues, and especially owing to the simplified model used while computing the steering vectors (we just used an equivalent homogeneous medium since the breast features are in general unknown), $\Phi_{I-MUSIC}$ results smeared and delocalized from the actual tumor position. Indeed, this is a drawback common to any radar approach that relies on an assumed scattering model. Nonetheless, this method is extremely quick and does not require a priori information about the used antenna, which was completely ignored in the imaging procedure [33,35]. Finally, we once again remark that the circular boundaries appearing in the reconstructions (i.e., panels (a) to (e)) just delimited the spatial region within which the reconstruction was carried out. The actual boundary of the breast was removed by the clutter rejection procedure. The relative (with respect to the image area) size of phantom coronal slice can be appreciated in panel (f) of Figure 6, where the boundary of the image spatial region has been overlapped to the phantom MRI. Eventually, the spots highlighted by the I-MUSIC does not in general indicate the actual tumor positions. However, they allows a clear tumor detection and to highlight in which quadrant (of the coronal slice) it appears.

**Figure 7:**
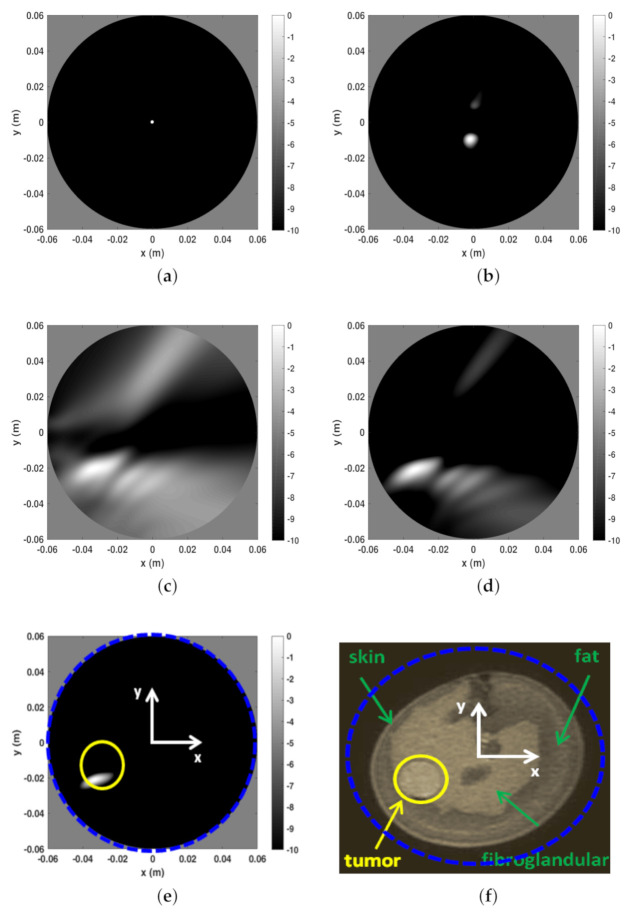
Reconstructions and MR image for phantom A. (**a**) Reconstruction with only time-gating, $N_{f}=20$. (**b**) Reconstruction with time-gating + rejection of the first two SVD projections of the scattering matrix, $N_{f}=20$ (**c**) Reconstruction with time-gating + sensor selection + rejection of the first two SVD projections of the scattering matrix, $N_{f}=20$. (**d**) Reconstruction with time-gating + sensor selection + rejection of the first two SVD projections of the scattering matrix, $N_{f}=40$. (**e**) Reconstruction with time-gating + sensor selection + rejection of the first two SVD projections of the scattering matrix, $N_{f}=100$. (**f**) MR coronal slice image of Phantom B. In particular, in panel (**f**) the blue dashed circle indicates the circular boundary of the spatial region within which the reconstructions reported in the other panels have been achieved. This is highlighted even in panel (**e**). Moreover, in the latter, the yellow circle denotes the tumor location and size.

The second reconstruction example refers to phantom B and is reported in Figure 7. In this case, the entropy procedure returned the same time-gating as above and almost the same observation angular coverage. Indeed, by considering four different slice heights, $n_{i}$ ranged from 28 to 34 while $n_{s}$ remained 56. The same discussion as above applies here, with the tumor being very well detected. However, we remark that since phantom B is more complex (from a morphological point of view) than phantom A (see panel (f)) and exhibits less “circular” symmetry, it is reasonable that clutter space dimension was increased. To this end, during the subspace projection clutter reduction stage the first two (instead of only the first one) projections of the scattering matrix were discarded in Equation (32). In general, it difficult to a priori set the number of projections to be discarded. Here, we used the conservative and heuristic approach to discard at most two projections. Rejecting more projections can help in further reducing clutter but the risk is that the tumor signal can be discarded as well.

## 5. Conclusions

Microwave breast imaging requires to deal with a number of issues which go far beyond the need do devise suitable inversion algorithms. Indeed, under the simplified linear framework subtended by the so-called radar approaches, which aim at a mere detection and localization of tumors, data must be properly pre-processed (before the imaging stage) to make sure that the resulting signals are actually useful to pursue the objective.

In this regard, one of the problems to be faced is the need to estimate or compensate for the antenna frequency response, especially when coherent wide band radar imaging methods are employed to obtain the scene image. Indeed, on one hand, antenna frequency response shapes the actually received pulse signals and modifies the overall round-trip delay; both these effects must be taken into account while implementing the beam-forming image procedure. On the other hand, because of the close proximity set-up usually adopted in breast imaging, the antenna couples with the unknown breast and its response becomes different from the free-space case. To overcome this drawback, it is shown that incoherent methods, that do not simultaneously use the frequency data but rather process each frequency separately and then combine the outcomes, can be employed with a minor reduction of the performance, especially if incoherence is used in conjunction to a MUSIC like algorithm (I-MUSIC).

Another crucial aspect is the clutter that overwhelms the target signals and can impair imaging. In this paper we have introduced a new multi-step clutter rejection method that is based on two entropy computations for time-gating setting and the selection of the sensors whose signal are less corrupted by clutter, followed by a standard subspace rejection procedure based on the SVD computation of the scattering data matrix.

The effectiveness of the de-clutter plus I-MUSIC has demonstrated against experimental data collected by using a multimodal phantom we previously developed and characterized in [33]. The results show that, for the considered phantoms, the proposed method very well succeed in detecting and localizing the tumor, though the dielectric contrast with respect to the surrounding fibroglandural tissue was only 1.2:1. This contribution can be considered as completing [33], where we mainly focused on the phantom manufacturing and characterization and only barely described the microwave imaging procedure.

As a concluding remark, we would like to remark that microwave breast imaging is a very broad research field and by this paper we did not intend to give a comprehensive account of the huge available literature. We have just focused the spot on our specific perspective.

## Data Availability

No new data were created or analyzed in this study. Data sharing is not applicable to this article.
